# Laparoscopic Treatment of Adrenal Tumors: A Single-Center Experience with 58 Patients

**DOI:** 10.1155/2016/9574391

**Published:** 2016-11-16

**Authors:** Aziz Ari, Kenan Buyukasik, Cihad Tatar, Ozgur Segmen, Feyzullah Ersoz, Soykan Arikan, Feray Gunver, Serkan Sari

**Affiliations:** ^1^Department of General Surgery, Istanbul Education and Research Hospital, Istanbul, Turkey; ^2^Department of Pathology, Istanbul Education and Research Hospital, Istanbul, Turkey

## Abstract

*Background.* The aim of this study is to discuss the laparoscopic approach and assess the immunohistochemical expression profiles of synaptophysin, Ki-67, and inhibin and patient outcomes in adrenal masses through a series of cases treated at our institution.* Method.* The study was conducted on 58 patients who were diagnosed with adrenal masses. All cases were operated on laparoscopically for adrenal masses.* Results.* Both inhibin and synaptophysin were found positive in 45 patients (77,6%). Ki-67 was negative in 11 patients, whereas it was found positive in 42 with a rate of 1%. The size of the masses ranged from 1 up to 9 cm (mean 4,3 ± 1,5). Urine hormone excretion was measured within normal ranges in 47 out of 58 patients (81%). Most of the diagnosed patients were harboring Cortical Adenoma (*n*: 38; 65,5%). All of the masses were successfully resected without complication except 3 patients. Because of complications of bleeding, the operation was converted to open surgery for 2 patients.* Conclusion.* Morbidity, mortality, and healing were comparable, regardless of tumor size, yet involvement in both laparoscopic and adrenal surgery was required. Our results suggested that laparoscopic adrenalectomy should replace open surgery as the standard treatment for most adrenal masses.

## 1. Introduction

Adrenal masses often happen in the peripheral layer of the organs, which is known as the adrenal cortex. For the most part, the frequency of adrenal tumors is much lower than that of different tumors in people, yet the uncommonness of these tumors should not negate their clinical criticalness in view of their specific areas and potential endocrine impacts.

When accurately analyzed and appropriately treated, most adrenal tumors are operable. Subsequently, laparoscopic surgery for adrenal mass has picked up prevalence, and a few organizations have extended signs for this technique [1]. Possibly threatening essential adrenal tumors and singular adrenal metastases, once considered contraindications for the laparoscopic methodology, are presently being uprooted laparoscopically in a few foci [2]. The suitability of the laparoscopic way to deal with essential adrenal carcinomas remains the center of level-headed discussions [3]. A healing laparoscopic resection consolidates the oncologic standards of the open strategy, maintaining a strategic distance from crack of the tumor container. Along these lines, there ought to be confirmation drawn by operational morbidity and mortality that laparoscopic surgery is just as protected or even more secure than traditional open surgery is [4].

When the pathogenesis and the awareness of the danger of adrenal masses are considered, it proceeds with huge regions of vulnerability [1]. A few histopathological and immunohistochemical markers have risen in the previous two decades that incorporate markers, for example, as steroidogenic factor-1 that might be valuable in building up the adrenocortical source of an adrenal mass, while different markers, for example, Ki-67 and p53, could stratify tumors into prognostic gatherings [5]. The aim of this study is to discuss the laparoscopic approach and assess the immunohistochemical expression profiles of synaptophysin, Ki-67, inhibin, and patient outcomes in adrenal masses through a series of cases treated at our institution in the context of a comprehensive relevant literature review.

## 2. Method

The study was conducted on 58 patients who were diagnosed with adrenal masses in Istanbul Education and Research Hospital. All cases were operated on for adrenal masses between the dates of January 2011 and November 2015. The data of the patients included history, clinical assessment, radiological studies, and management and pathology of the excised adrenal masses. All patients were routinely investigated by plain radiography and ultrasonography. Other radiological studies such as enhanced abdominal computed tomography scan, abdominal magnetic resonance imaging (MRI) study, intravenous urography, and angiography were requested according to availability and case specification.

All cases aimed to be managed by laparoscopic adrenalectomy. The cases whose adrenal hormones were active gained drug for alpha-adrenergic blockade at least 14 days until being normotensive and then were operated on. Indications for adrenalectomy included functioning adrenal masses, pain, suspicious masses for malignancy, or incidentalome. Intraoperative complications included hemorrhage and vascular or abdominal injuries, while postoperative complications included secondary hemorrhage, bronchopneumonia, and prolonged ileus.

## 3. Results

The number of the patients diagnosed with adrenal masses admitted to our hospital increased in the last four years as of November 2015. Totally, 58 patients (30 males/28 females) were diagnosed to have adrenal masses. The ages of the patients ranged from 21 to 72 years (mean: 49,8 ± 11,3). The distribution of the masses was as 35 right-sided and 23 left-sided. Both inhibin and synaptophysin were found positive in 45 patients (77,6%). Ki-67 was negative in 11 patients, whereas it was found positive in 42 with a rate of 1%. In 4 patients, Ki-67 was at 1%-2%, 2%, 2%-3%, and 3%-4% rates in one each. The size of the masses ranged from 1 cm up to 9 cm (mean 4,3 ± 1,5). The dimensions were as 1 cm in 3 patients (5,2%), 2 cm in 2 patients (3,4%), 3 cm in 9 patients (15,5%), 4 cm in 21 patients (36,2%), 5 cm in 12 patients (20,7%), 6 cm in 7 patients (12,1%), 7 cm in 2 patients (3,4%), 8 cm in 1 patient, and 9 cm in another patient. The demographic data and the tumor characteristics are given at Table 1.

Urine hormone excretion was measured within normal ranges in 47 out of 58 (81%). Similarly, venous hormone levels were found as regular in 48 patients. Most of the diagnosed patients were harboring Cortical Adenoma (*n*: 38; 65,5%) (Table 2). Other masses were pheochromocytoma (*n*: 9; 15,5%), mucinous adenocarcinoma (*n*: 2; 3,4%), benign cystic formation (*n*: 2; 3,4%), myelolipoma (*n*: 2; 3,4%), adenocarcinoma infiltration (*n*: 2; 3,4%), adrenocortical tumor (*n*: 1; 1,7%), adrenal lipoma (*n*: 1; 1,7%), and suprarenal cyst (*n*: 1; 1,7%) (Table 3). Screening by plain radiography showed apparently disease-free evidence in most patients, but 8 (13.8%) showed evidence of calcification and/or soft tissue mass. USG could diagnose the presence of masses in 47 patients (81%).

Before the operations, alpha-adrenergic blockade medication was applied to the patients whose adrenal hormones were active in 14 days until it became normotensive. Excision of the diagnosed masses with transperitoneal laparoscopic approach was offered to all 58 patients. All of the masses were successfully resected without complication except 3 patients. During operation of these 3 patients, suprarenal venous vessel was injured as a result of the procedure. Because of complications of bleeding, we started open surgery for 2 patients. The average operative time ranged between 55 and 190 minutes. The bloody fluid had a volume of 150–750 mL from the drains.

## 4. Discussion

This study is a review of our involvement with laparoscopic adrenalectomy with 58 cases over a time of 5 years. By the time, laparoscopic adrenalectomy has turned into the highest quality level in administration of the most adrenal masses [6]. Actually, in the course of the most recent decade, in retrospective comparison studies it has been reported that the laparoscopic approach is being used more than the routine open surgery methodology for the evacuation of benign functioning and nonfunctioning neoplasms of the adrenals [7]. By assessing these 58 cases treated at our institution, we revealed that laparoscopic methods are connected with diminished hospitalization time; lessened operative blood loss; less postoperative inconvenience, pain, and requirement for analgesics; speedier postoperative recuperation; earlier returning to ordinary exercises and eating regimen; and lower general costs [8]. Taking into account these contemplations, the signs for this method have been incomprehensibly extended, and laparoscopic adrenalectomy might even be performed in selected cases, on an outpatient premise. Our results will contribute the positive and low-risk outcomes of laparoscopic approaches in the literature.

Adrenal organ tumors require a multidisciplinary approach and methodology [9]. In the literature, the mean age of an individual determined to have an adrenal tumor is somewhere around 45 and 50 [10]. In our cases, the ages of the patients ranged from 21 to 72 years (49,8 ± 11,3). In any case, these tumors can emerge at any age of lifetime. A greater number of females than males have a tendency to be determined to have adrenal tumors [1]. Our study varies from this perception. The number of males and females with adrenal masses was comparative in the present study. One reason is that men are embarrassed to go to clinics for the treatment of such a disease in our society.

Customarily, open surgical approaches to the adrenal mass were the standard of consideration [7]. In the most recent decade, laparoscopic methodology was initially applied for low-sized adrenal masses until the technique turned out to be well established [11]. At that point, indications reached out to include bigger ones. Strong adrenal masses up to 10 cm and adrenal growths up to 16 cm in distance across could be effectively overseen [10]. There is no cut-off level with respect to the extent of the adrenal mass; however there are numerous reports about effective laparoscopic adrenalectomy for strong masses up to 14 cm in their biggest breadth [6]. In our institute, the open approach is preferred for adrenal lesions suspicious of malignancy, especially those of large size with signs of local infiltration or venous involvement. Otherwise, we choose laparoscopic adrenalectomy for adrenal masses, unless a damaging complication exists [12]. In these cases, all masses were successfully resected without complications except 3 patients. During the operations of these 3 patients, suprarenal venous vessel was injured as a result of the procedure. Because of difficulties of bleeding, we started open surgery for 2 patients.

The role of laparoscopy in the case of adrenal mass is still easily proven wrong due to neighborhood repeat and port-site metastases [7]. Moinzadeh and Gill [11] conducted a study with 31 patients with adrenal malignancies by laparoscopic adrenalectomy. Within 26 months, only 42% of the patients survived as free of the disease. Porpiglia et al. [9] reported a high frequency of neighborhood repeat or port-site seeding after laparoscopic adrenalectomy for threatening injuries. In a case series of Gomha et al. [13], investigating the treatment of adrenal tumors with 238 cases, 5 masses of adrenal harm were overseen laparoscopically. Three masses were confined metastases from bladder, liver, and bosom growths while 2 were essential adrenal tumor. Local recurrence and death were encountered during the first year of follow-up in all the patients. In our case series, we operated on all masses with laparoscopic approach. Of 58 cases, we only encountered two isolated metastases one originating from lung tissue and another from renal cell carcinoma metastases from right renal tissue.

A few authors have thought about open or laparoscopic approaches reflectively and presumed that laparoscopic adrenalectomy is prevalent as far as intraoperative blood loss, transfusion rates, fast oral intake, and absence of pain necessities [1, 6, 14]. Although long operative time was frequently reported by Yoshimura et al. in the early involvement with laparoscopic adrenalectomy [15], presently, it is evident that the strategy gives a factually critical shorter operation time [14]. Also, laparoscopic adrenalectomy has been ended up to be superior in both pediatric and morbid obese cases [16]. Gomha et al. [13] uncovered an essentially shorter healing center stay with matched complication rate in the laparoscopic approach regardless of being used in smaller-sized adrenal masses. In our study, supporting and similar to the literature, the average operative time was around 80 minutes that ranged between 55 and 190 minutes.

While adrenal inhibins are not stained in* zona glomerulosa*, they are stained weakly in* zona fasciculate* and strongly in adrenal cortex and* zona reticularis* [17]. For this reason, adrenal inhibin expression is important in distinction between adrenal-originated tumors. It was reported in studies conducted on the same topic that all of the adrenal tumors had an immunoreactivity against adrenal inhibin antibody [18]; however, in some other studies conducted later, it was shown that adrenal tumors might sometimes be immunonegative, and this situation would not exclude adrenocortical carcinoma; and in addition to this, it was also shown that it could not be used in distinguishing between benign and malign [17, 19]. Renshaw and Granter [20] conducted a study and reported that inhibin and synaptophysin positivity could be used in definitive diagnosis of adrenocortical carcinoma. In our study, inhibin and synaptophysin positivity was detected in 45 (77,6%) patients.

In some published studies it was reported that the immunohistochemical analysis of Ki-67, which is the marker of cellular proliferation, is a more reliable method in the classification of the diagnosis and prognosis of adrenal tumors than the mitotic index [21–23]. In our study, Ki-67 was detected as negative in 11 (19%) patients, while it was determined that the Ki-67 expression increased in 47 (81%) of the patients. Although Ki-67 expression and inhibin and synaptophysin positivity are used in supportive markers in adrenal tumors, they are not associated with malignity on their own.

## 5. Conclusion

Today, pheochromocytoma and neuroblastoma together with adrenocortical adenoma are the most regularly diagnosed pathologies, as we see in our foundation. Appropriate and provoke administration of adrenal masses results in sensible morbidity and minimum mortality. Although Ki-67 expression and inhibin and synaptophysin positivity are used as supportive markers in adrenal tumors, they are not associated with malignity on their own. Laparoscopic adrenalectomy can be viewed as the treatment of decision for every adrenal mass up to 9 cm as we encountered and proposed. Morbidity, mortality, and healing facility stay were comparable, regardless of tumor size, yet involvement in both laparoscopic and adrenal surgery is required. Extensive sized tumors suspected of being a primary malignancy based on imaging characteristics might be approached with the open technique from the start, if laparoscopy has contraindications. Our results suggested that laparoscopic adrenalectomy should replace open surgery as the standard treatment for most diagnosed adrenal masses.

## Figures and Tables

**Table 1:** Demographics and tumor characteristics (*n*: 58).

Mean age, mean (range)	49,8 ± 11,3 (21–77)
Male/female, *n*	30/28
BMI, median (range)	27.8 kg/m^2^ (18,1–40)
Side (left/right), *n*	23/35
Ki67 positivity, *n* (%)	41 (70%)
Inhibin positivity, *n* (%)	45 (77,5%)
Synaptophysin positivity, *n* (%)	45 (77,5%)
Mean tumor size, mean (range)	4,3 ± 1,5 cm (1–9)

**Table 2:** Postoperational outcomes of all operated cases.

Operating time, median (range)	80 min (55–190)
Estimated blood loss, median (range)	150 mL (100–600)
Regular urine hormone, *n* (%)	47 (81%)
Regular venous hormone, *n* (%)	48 (82.7%)
Complications, *n* (%)	3 (5.1%)
Major complications, *n* (%)	2 (3.4%)
Mortality, *n*	None

**Table 3:** Pathologic types of all excised adrenal masses.

Tumor types	*n*	%
Cortical Adenoma	38	65,5
Pheochromocytoma	9	15,5
Adenocarcinoma Infiltration	2	3,4
Benign cystic formation	2	3,4
Mucinous adenocarcinoma	2	3,4
Myelolipoma	2	3,4
Adrenocortical *T*_*m*_	1	1,7
Adrenal lipoma	1	1,7
Suprarenal cyst	1	1,7
